# Anti-COVID-19 activity and simple HPLC method for concurrent detection of repurposed drugs in novel binary mixtures

**DOI:** 10.1186/s13568-026-02030-8

**Published:** 2026-04-09

**Authors:** Mohamed A. Abd elsalam, Omnia Kutkat, Yassmin Moatasim, Ahmed Mostafa, Mohamed Gaballah, Alaa-eldin Elgendy, Mohamed A Ali, Ghada Hadad, Sarah Shabayek

**Affiliations:** 1 Department of Pharmaceutical Analytical Chemistry, Faculty of Pharmacy, Suez Canal University, Ismailia, Egypt; 2 The Center of Scientific Excellence for Influenza Viruses, Department of Water Pollution Research, Environmental Research Institute, National Research Center, Giza, Egypt; 3 Department of Microbiology and Immunology, Faculty of Pharmacy, Suez Canal University, Ismailia, 41522 Egypt

**Keywords:** COVID-19, Antiviral, HPLC, Remdesivir, Nitazoxanide, Daclatasvir, Piroxicam

## Abstract

The discovery of a new antiviral drug specifically targeting SARS-CoV-2 would require considerable time and effort. Repositioning drugs that are already used for other viral infections is a valuable approach. Remdesivir is the first antiviral treatment approved by the Food and Drug Administration (FDA) for COVID-19. Nitazoxanide is a broad-spectrum anti-infective agent that has been suggested for repurposing in COVID-19 treatment. Recent research has demonstrated the synergistic anti-SARS-CoV-2 antiviral activity of Remdesivir when combined with Nitazoxanide. Moreover, Daclatasvir is an FDA-approved antiviral for managing chronic HCV infection, and its potential use in treating COVID-19 has been reported. Piroxicam is an FDA-approved anti-inflammatory with some clinical evidence supporting its application for COVID-19. There is no evidence for exploiting the anti-SARS-CoV-2 activity of Daclatasvir when combined with Piroxicam. We were able to demonstrate the enhanced anti-SARS-CoV-2 activity of the Remdesivir-Nitazoxanide and Daclatasvir-Piroxicam binary mixtures in Vero-E6 cells. In addition, we were able to develop a simple HPLC methodology for the concurrent determination of the two analytes of each binary mixture (Remdesivir-Nitazoxanide mixture and Daclatasvir-Piroxicam mixture) in both pure form and in human plasma using non-complicated, widely available analytical instruments. The proposed method is simple, accurate, quick, and sensitive. No drug interactions were observed in the tested binary mixtures.

**Supplementary Information:**

The online version contains supplementary material available at 10.1186/s13568-026-02030-8.

## Introduction

The coronavirus disease (COVID-19) pandemic, which originated in China in December 2019, was caused by the novel severe acute respiratory syndrome coronavirus 2 (SARS-CoV-2). As a result, COVID-19 has become the most significant public health crisis of the century, with over 36 million confirmed cases globally, as of October 2020 (Sharma et al. 2021). Due to the urgent need for effective and safe treatment, researchers have initiated clinical trials to assess the efficacy of various medications. However, the discovery of a new antiviral drug specifically targeting SARS-CoV-2 would require considerable time and effort. Consequently, drugs already used for other viral infections have been repurposed for use (Ng et al. 2021; De et al. 2022; Krishnamurthy et al. 2022).

Remdesivir is a broad-spectrum antiviral nucleotide analogue prodrug that has gained Food and Drug Administration (FDA) approval as the first antiviral treatment for COVID-19. It primarily works through blocking viral RNA-dependent RNA polymerase, which results in the early termination of viral RNA synthesis (Radoshitzky et al. 2023). Preclinical research has shown that it is highly effective against various RNA viruses, including coronaviruses like SARS-CoV, SARS-CoV-2, and Middle East respiratory syndrome coronavirus (MERS-CoV), as well as filoviruses and paramyxoviruses (Radoshitzky et al. 2023). Nitazoxanide, a derivative of nitrothiazole benzamide, is an FDA-approved antiparasitic drug that is effective against a range of protozoa and helminths (Lokhande and Devarajan 2021). In addition, several clinical studies have confirmed the efficacy of Nitazoxanide in treating hepatitis B virus (HBV) and hepatitis C virus (HCV) infections (Lokhande and Devarajan 2021). It is reported to possess broad-spectrum antiviral properties against numerous viral diseases. It is now acknowledged for its wide-ranging activity against protozoa, helminths, bacteria, and a diverse array of viruses, such as influenza, norovirus, HBV, HCV, and coronaviruses (Lokhande and Devarajan 2021). Furthermore, it demonstrated significant in vitro effectiveness against SARS-CoV-2 in cell culture studies, suggesting its potential for repurposing in COVID-19 treatment (Lokhande and Devarajan 2021). Moreover, recent research demonstrated the synergistic anti-SARS-CoV-2 antiviral activity of Remdesivir when combined with Nitazoxanide (Bobrowski et al. 2021; Stewart 2023).

Daclatasvir is a first-in-class direct-acting antiviral (DAA). It is an FDA-approved drug that plays a crucial role in managing chronic HCV infection by targeting the nonstructural viral protein 5 A (NS5A). It has shown potent, pangenotypic antiviral activity and high rates of sustained virologic response (SVR) (Smith et al. 2016; Goel et al. 2019; Elbaz et al. 2025). Additionally, recent studies have investigated its potential use in treating COVID-19 (Bansode et al. 2023; Mattos et al. 2024). Piroxicam is an FDA-approved nonsteroidal anti-inflammatory drug (NSAID). Although major clinical guidelines do not currently endorse its use for the treatment of COVID-19 (Wojcieszyńska et al. 2022; Perico et al. 2023), there is some clinical evidence that supports its application for this condition (Ricciotti et al. 2021). Piroxicam was reported to significantly reduce the Angiotensin-converting enzyme 2 (ACE2) expression (Ricciotti et al. 2021; Perico et al. 2023) which acts as a primary entry point for SARS-CoV-2 to inter and infect human cells (Beyerstedt et al. 2021; Perico et al. 2023). In addition, Piroxicam was reported as an effective medication in controlling fever and cytokine storm with rapid improvement of oxygen saturation in COVID-19 patients (Varodi et al. 2022). For Daclatasvir and Piroxicam, there is no documented direct drug interaction between the two drugs. The safety profile and drug interaction risk of Daclatasvir have been well-studied, mainly involving antivirals and other medications commonly co-prescribed in HCV treatment (Garimella et al. 2016). Piroxicam is primarily used for pain and inflammation (Dahl and Ward 1982; Saganuwan 2016) and does not have documented interactions with Daclatasvir in the current pharmacological literature.

Several chromatographic analytical methods have been exploited for the determination of Remdesivir, Nitazoxanide, Daclatasvir, and Piroxicam in different matrices in pure forms (Yritia et al. 1999; Calvo et al. 2016; Avataneo et al. 2020; Almahri and Abdel-Lateef 2021; Nguyen et al. 2021; Xiao et al. 2021; Chauhan et al. 2025) or in combination with other drugs (Sharma et al. 2011; Sultana et al. 2013; Fayed et al. 2021, 2022; Habler et al. 2021; Bozhanov et al. 2024; El-Shorbagy et al. 2024; Ahmed et al. 2025a, b; Youssef et al. 2025). However, to the best of our knowledge, there is limited evidence for the simultaneous determination of a binary combination of Remdesivir and Nitazoxanide mixture or a binary combination of Daclatasvir and Piroxicam mixture using simple non-complicated high-performance liquid chromatography (HPLC) methodology and widely available analytical instruments. Hence, the current study aimed to determine the anti-SARS-CoV-2 activity and to develop a simple HPLC methodology for the concurrent determination of Remdesivir-Nitazoxanide and Daclatasvir-Piroxicam combination therapies for the management of COVID-19. The proposed HPLC method can be used for concurrent detection of the two analytes of each binary mixture (Remdesivir-Nitazoxanide mixture and Daclatasvir-Piroxicam mixture) in both pure form and in human plasma using non-complicated, widely available analytical instruments. The proposed method is simple, accurate, quick, and sensitive.

## Materials and methods

### Chemicals and reagents

HPLC-grade water (Sigma-Aldrich, St. Louis, Germany), Methanol (HPLC grade, Fisher Scientific, Germany), HPLC-grade Acetonitrile (Sigma-Aldrich, St. Louis, Germany), Trifluoracetic acid analytical grade (Sigma-Aldrich, St. Louis, Germany), and Orthophosphoric acid analytical grade (Sigma-Aldrich, St. Louis, Germany) were utilized. Human plasma was collected from healthy volunteers. The tested FDA-approved drugs, listed in Table 1, were kindly granted by the Egyptian International Pharmaceutical Industries “EIPICO”, the Holding Company for Pharmaceuticals, Chemicals, and Medical Appliances “HoldiPharma”, and the National Organization for Drug Control and Research in Egypt.

**Table 1:** FDA-approved drugs evaluated in the current study against SARS-CoV-2 (NRC-03-nhCoV), including dosage forms, labeled strengths, and reference standard purities

FDA-approved drug	Therapeutic indication	Pharmaceutical dosage form used	Labelled strength	Reference standard purity (% CoA)
Remdesivir	Antiviral	Injectable vial (lyophilized powder)	100 mg/vial	≥ 99.0
Daclatasvir	Antiviral	Film-coated tablet	60 mg/tablet	≥ 98.5
Nitazoxanide	Broad-spectrum antimicrobial	Film-coated tablet	500 mg/tablet	≥ 99.0
Niclosamide	Anthelmintic/ antibacterial	Tablet	500 mg/tablet	≥ 98.0
Azithromycin	Antibacterial	Film-coated tablet	500 mg/tablet	≥ 98.0
Doxycycline	Antibacterial	Capsule	100 mg/capsule	≥ 98.0
Piroxicam	Nonsteroidal anti-inflammatory	Tablet	10 mg/tablet	≥ 99.0
Hydroxychloroquine	Antimalarial	Film-coated tablet	200 mg/tablet	≥ 98.0
Riboflavin (Vitamin B2)	Vitamin	Tablet	10 mg/tablet	≥ 99.0
Cholecalciferol (Vitamin D3)	Vitamin	Capsule	1000 IU/capsule	≥ 99.0

## Vero-E6 cells and SARS-CoV-2 virus

Vero-E6 cells were maintained in Dulbecco’s Modified Eagle’s medium (DMEM) containing 10% Fetal Bovine Serum (FBS) (Invitrogen) and 1% Penicillin/Streptomycin (pen/strep) antibiotic mixture at 37 ◦C, 5% CO2. All the experiments with SARS-CoV-2 virus were conducted at a certified biosafety level 3 facility at Center of Scientific Excellence for Influenza Viruses, Vaccine Development and Virological Tests Unit, National Research Centre, Giza, Egypt. Passage 2 (P2) virus stock was propagated in Vero E6 cells that were distributed into tissue culture flasks (3 × 10^5^ cells/mL) 48 h before infection with hCoV-19/Egypt/NRC-3/2020 isolate at a multiplicity of infection (MOI) of 0.1 in infection medium (DMEM containing 2% FBS, 2% pen/strep, and 1% L-1-tosylamido-2-phenylethyl chloromethyl ketone (TPCK)-treated trypsin (to enhance viral entry and propagation in cell culture). Two hours later, the infection medium containing the virus inoculum was removed and replaced with fresh infection medium and incubated for three days. At the indicated time point, cell supernatant was collected and centrifuged for 5 min at 2500 rpm to remove small particulate cell debris. Transferring the supernatant to a new 50 mL Falcon tube, aliquoting it, and titrating it with the plaque titration assay (Mendoza et al. 2020) was carried out.

## Plaque titration assay

To define the countable viral titer of virus (Mendoza et al. 2020), in 6 well plates Vero-E6 cells were distributed in Dulbecco’s Modified Eagle’s Medium (DMEM) (DMEM; Bio Whittaker, Walkersville, MD, USA) supplemented with fetal bovine serum (FBS) (5%) (Gibco-BRL; New York, USA) and penicillin/streptomycin (pen/strep) antibiotic/antimycotic mixture (2%) (GIBCO-BRL; New York, USA) and incubated overnight in a humidified 37 ◦C incubator under 5% CO_2_ condition. The cell monolayers were then washed once with 1× PBS and subjected to virus adsorption for 1 h in a humidified 37 ◦C incubator under 5% CO_2_ conditions after ten-fold dilution. Inoculum was removed from each well, and over layer was added with 50% agarose (2x) and 50% (2x) medium after solidification. The plates were then incubated for 72 h. The plate was fixed with 10% formaldehyde and stained with 0.1% crystal violet solution to visualize the viral plaques. The following formula was used to determine the viral titer:

Plaque-forming unit (PFU/mL) = Number of Plaques X Reciprocal of virus dilution X Dilution Factor (to 1mL).

## Cytotoxicity assay

The crystal violet assay was employed as previously described in Kutkat et al. 2022 (Kutkat et al. 2022) to determine the half maximum cytotoxic concentration _50_ (CC_50_) on Vero-E6 cells for each mixture of drugs. Briefly, Vero-E6 cells were cultivated in cell culture plates (100 µL/well at a density of 3 × 10^5^ cells/mL) and incubated for 24 h in a humidified 37 ◦C incubator under 5% CO_2_ conditions. Next, the plates were washed with sterile 1x PBS, and successive two-fold serial dilutions in the range of 1000 to 1 µM of the investigated drugs/mixtures were added to the cultured wells in triplicate, including the untreated cell control wells. Plates were then incubated at 37 °C under 5% CO_2_ in humidified conditions for 3 days to assess the CC_50_ for each compound. After the incubation period, the cell monolayers were fixed with 10% formaldehyde and stained with 0.1% crystal violet. Finally, the crystal violet stain in dried plates was dissolved by adding 180 µL of absolute methanol. The optical density (OD) was measured at a wavelength of 570 nm using an ELISA plate reader (Anthos Labtec Instruments, Heerhugowaard, Netherlands). Using GraphPad prism (version 8.02), the cytotoxicity % compared to untreated cells was calculated by plotting cell viability % and viral inhibition % versus the concentration of each drug/mixture.

## Half maximal inhibitory concentrations 50 (IC_50_)

The crystal violet assay (Kutkat et al. 2022) was used to determine the half maximal inhibitory concentrations 50 (IC_50_) for each drug mixture. Briefly, Vero-E6 cells were distributed in 96-well tissue culture plates and incubated overnight at 37 °C in a humidified incubator under 5% CO_2_ conditions. The cell monolayers were then washed once with 1× PBS and subjected to virus adsorption, tissue culture infection dose-50 (TCID50) of 100 (approximately corresponding to a MOI of 0.001), for 1 h at room temperature. The cell monolayers were further overlaid with 100 µL of 1x DMEM containing varying safe concentrations of the test compounds (less than CC_50_), including untreated cell control wells and virus-infected untreated cells as a virus control for normalization. After 72 h of incubation at 37 °C in a 5% CO_2_ incubator, the cells were fixed with 100 µL of 4% paraformaldehyde for 2 h and stained with 0.1% crystal violet in distilled water for 15 min at room temperature. The crystal violet dye was then dissolved using 180 µL absolute methanol per well, and the optical density of the color was measured at 570 nm using an ELISA reader (Anthos Labtec Instruments, Heerhugowaard, Netherlands). The IC50 of the compound is required to reduce the virus-induced cytopathic effect (CPE) by 50%, relative to the virus control. Using GraphPad prism (version 8.02), IC_50_ values were calculated using nonlinear regression analysis by plotting log inhibitor versus normalized response (variable slope).

### Safety index (SI) calculation

The safety index represents the ratio of CC_50_ to IC_50_. Higher SI values indicate a safer and more effective window for the investigated drug. The SI value was calculated using the following formula: SI = CC_50_/IC_50_.

## HPLC procedure

### Instrumentation

Chromatographic analysis was performed on a Waters Alliance 2690 HPLC system (Waters Corporation, Milford, MA, USA) comprising an integrated quaternary pump, autosampler, and column oven, and coupled to a Waters 996 photodiode array (PDA) detector (Waters Corporation, Milford, MA, USA).

### Standard stock solutions

Primary stock solutions of Remdesivir, Nitazoxanide, Daclatasvir, and Piroxicam were prepared separately in methanol at a concentration of 1 mg/mL (1000 µg/mL). Appropriate working solutions were obtained by serially diluting stock solutions with methanol to cover the required concentration ranges for analysis. Mixed working solutions of Remdesivir–Nitazoxanide and Daclatasvir–Piroxicam were prepared at suitable concentrations and evaporated to dryness under a gentle stream of nitrogen. The residues were reconstituted in 120 µL of the mobile phase, filtered through a 0.22 μm nylon syringe filter, and 100 µL was injected into the HPLC system.

### Plasma preparations

Primary stock solutions of Remdesivir, Nitazoxanide, Daclatasvir, and Piroxicam were prepared separately in methanol at a concentration of 1 mg/mL. Appropriate working solutions were obtained by serially diluting the stock solutions with methanol. Calibration standards were prepared by spiking blank human plasma with the corresponding working solutions to yield final concentrations of 1000, 800, 600, 400, 200, and 100 ng/mL for the Remdesivir–Nitazoxanide mixture and 600, 500, 400, 300, 200, and 100 ng/mL for the Daclatasvir–Piroxicam mixture. Blank plasma used for calibration and validation was obtained from six independent sources to ensure selectivity toward endogenous matrix components.

Quality control (QC) samples were independently prepared in plasma at four concentration levels in accordance with ICH M10 bioanalytical validation guidelines (Guideline 2022b). For the Remdesivir–Nitazoxanide mixture, the QC levels were Lower Limit of Quantification (LLOQ) QC (100 ng/mL), Low Quality Control (LQC) (200 ng/mL), Mid Quality Control (MQC) (400 ng/mL), and High Quality Control (HQC) (800 ng/mL), while for the Daclatasvir–Piroxicam mixture they were LLOQ QC (100 ng/mL), LQC (200 ng/mL), MQC (300 ng/mL), and HQC (500 ng/mL). The LLOQ was experimentally validated in plasma and met the acceptance criteria for accuracy (± 20%) and precision (≤ 20% RSD).

Plasma calibration standards and QC samples were processed using protein precipitation by the addition of acidified acetonitrile (1% acetic acid), followed by centrifugation at 15,000 rpm for 15 min at 4 °C. The clear supernatant was collected and evaporated to dryness under a gentle stream of nitrogen. The residue was reconstituted in 120 µL of the mobile phase, filtered through a 0.22 μm syringe filter, and 100 µL was injected into the HPLC system.

Calibration curves in plasma were constructed using weighted linear regression (1/x) to compensate for concentration-dependent variance and to ensure accurate quantification across the calibration range, particularly at lower concentrations. Chromatographic analysis was performed immediately after preparation and after 24 and 48 h of storage at ambient autosampler temperature (25 °C) to evaluate short-term autosampler stability.

### Pharmaceutical dosage form preparations

Commercial Nitazoxanide tablets (500 mg/tablet) were finely powdered, and an accurately weighed portion equivalent to 100 mg Nitazoxanide was transferred into a 100 mL volumetric flask, dissolved in methanol, and sonicated for 15 min to ensure complete dissolution due to its limited aqueous solubility. The solution was diluted to volume with methanol, centrifuged at 3000 rpm for 10 min, and filtered through a 0.22 μm syringe filter to obtain a 100 µg/mL solution.

For daclatasvir tablets (60 mg/tablet) and piroxicam tablets (10 mg/tablet), accurately weighed powdered portions equivalent to the labelled amounts were transferred separately into 100 mL volumetric flasks, dissolved in 20 mL methanol, sonicated for 10 min, and diluted to volume with water to obtain final concentrations of 600 µg/mL and 100 µg/mL, respectively. The solutions were centrifuged at 3000 rpm for 10 min and filtered through 0.22 μm syringe filters before analysis.

The Remdesivir injectable vial (100 mg/vial) was dissolved in 5 mL of methanol, sonicated until complete dissolution, transferred to a 100 mL volumetric flask, and diluted to volume with water to obtain a 1000 µg/mL solution, followed by centrifugation and filtration.

A uniform injection volume (100 µL) was applied for all reference standards and pharmaceutical dosage form samples, and its suitability was confirmed during method validation.

### Chromatographic procedure

Samples were injected onto a C18 column (4.5 × 150 mm, 3.6 μm) using a Waters Alliance 2690 HPLC system equipped with a Waters 996 photodiode array detector. Isocratic elution was employed for the Remdesivir–Nitazoxanide mixture, whereas gradient elution was applied for the Daclatasvir–Piroxicam mixture (Table 2).

**Table 2:** Gradient elution of the mobile phase for detection and quantitation of Dalactasvir-Piroxicam mixture

Time (min)	Aqueous phase%	Acetonitrile%
0	85	15
10	50	50
15	85	15

The mobile phase for Remdesivir, Nitazoxanide, and their mixture consisted of 0.1% trifluoroacetic acid in water: acetonitrile (60:40, v/v). For Daclatasvir, Piroxicam, and their mixture, the mobile phase consisted of 0.1% orthophosphoric acid in water: acetonitrile (v/v) using the gradient program described in Table 2. The flow rate was maintained at 1.5 mL/min at ambient temperature, and the total run time was 15 min.

Quantitation was performed using UV detection at 290 nm for the Remdesivir–Nitazoxanide mixture and 320 nm for the Daclatasvir–Piroxicam mixture.

## Results

### Anti-SARS-CoV-2 activity

**Fig. 1:**
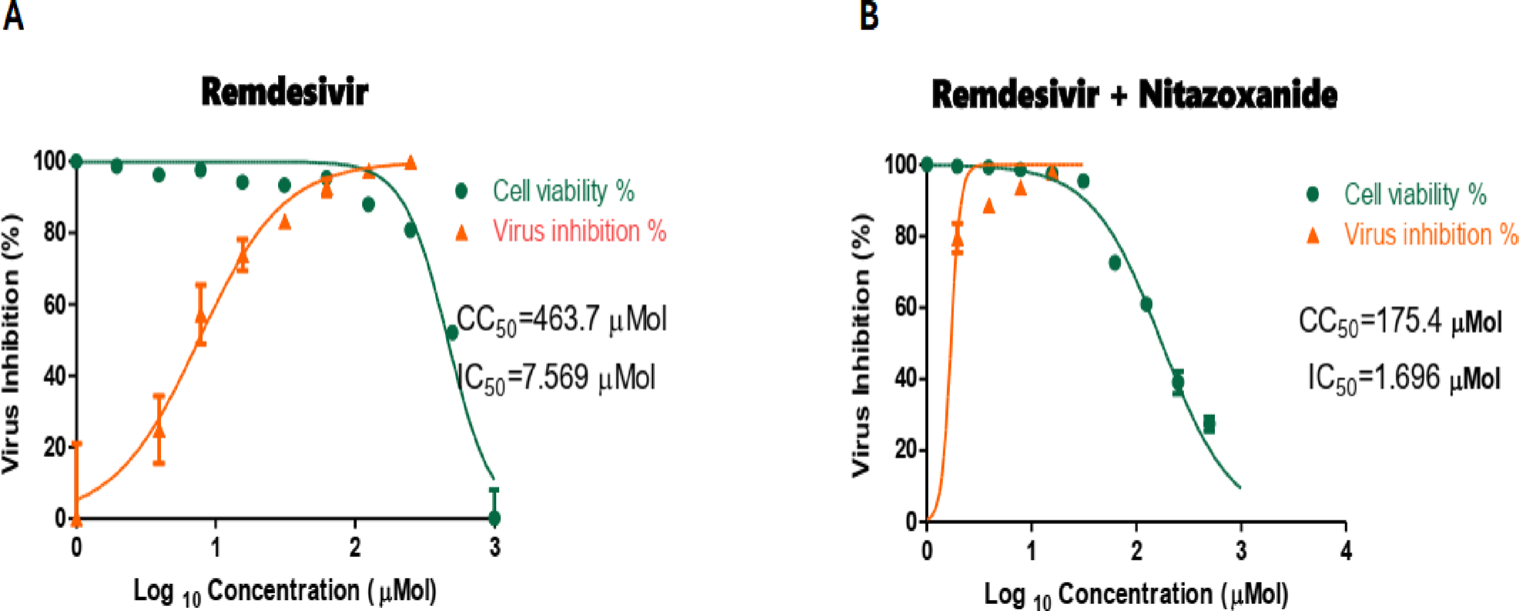
Cytotoxicity and Inhibition Curves for **A**, Remdesivir, and **B**, Remdesivir-Nitazoxanide mixture against NRC-03-nhCoV. Cytotoxic concentration (CC_50_) and Inhibitory concentration 50% (IC_50_) values were calculated using nonlinear regression analysis of Graph Pad Prism Software. (version 8.02) by plotting log inhibition versus normalized response (variable slope)

**Fig. 2:**
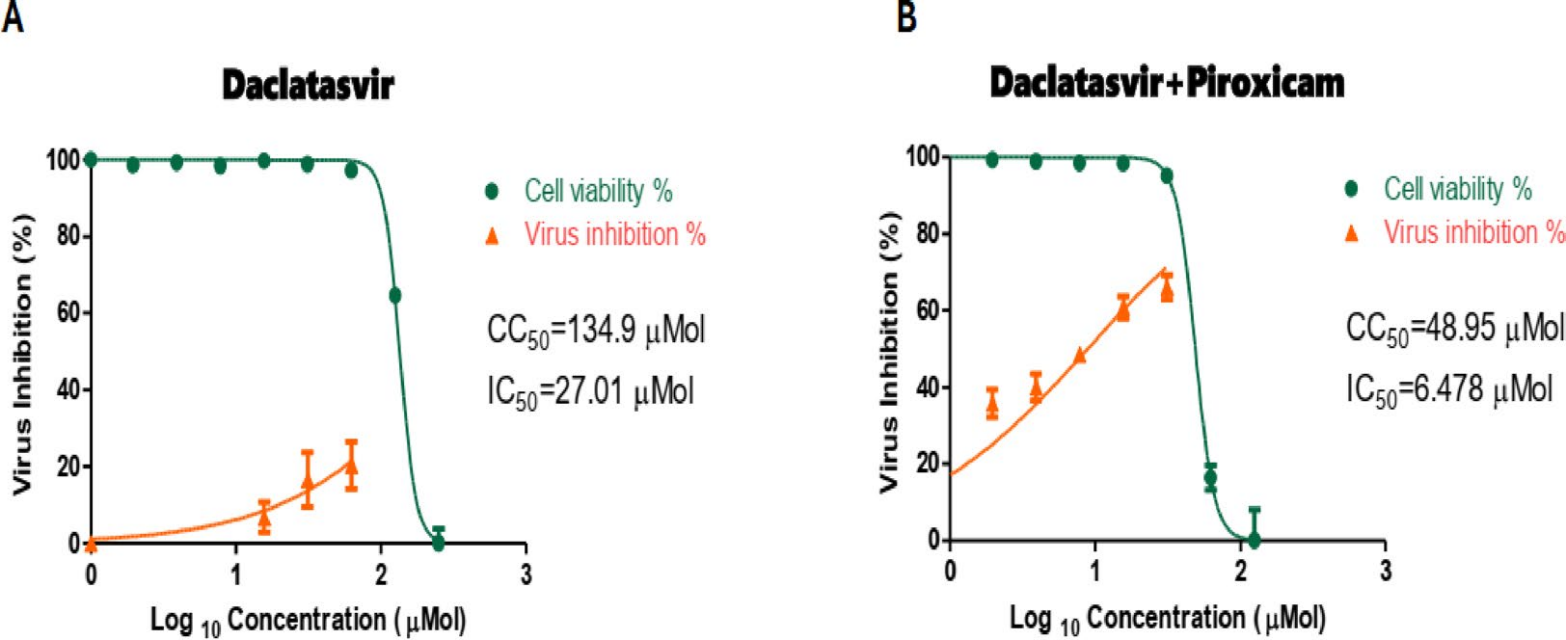
Cytotoxicity and Inhibition Curves for **A**, Daclatasvir, and **B**, Daclatasvir-Piroxicam mixture against NRC-03-nhCoV. Cytotoxic concentration (CC_50_) and Inhibitory concentration 50% (IC_50_) values were calculated using nonlinear regression analysis of Graph Pad Prism Software. (version 8.02) by plotting log inhibition versus normalized response (variable slope)

Enhanced anti-SARS-CoV-2 activity was observed for both the Remdesivir-Nitazoxanide and Daclatasvir-Piroxicam mixtures. Detailed IC_50_, CC_50_, and SI values are shown in Table 3; Figs. 1, 2. After demonstrating the anti-SARS-CoV-2 activity of the Remdesivir-Nitazoxanide and Daclatasvir-Piroxicam mixtures, as revealed by IC_50_, these mixtures were submitted for further HPLC analysis to detect potential drug interactions in solvent and plasma. No drug interactions were observed in the binary mixtures tested.

**Table 3:** Antiviral activity of tested FDA-approved antiviral drugs alone and in combination with FDA-approved antimicrobial and anti-inflammatory agents against SARS-CoV-2 (NRC-03-nhCoV) strain

FDA-approved drug/mixture	CC_50_ (Vero-E6) (µM)	IC_50_ (NRC-03-nhCoV) (µM)	SI (CC_50_ / IC_50_)
Remdesivir alone	463.70	7.57	61.25
Remdesivir + nitazoxanide	175.40	1.69	103.78
Daclatasvir alone	143.90	27.01	5.32
Daclatasvir + piroxicam	48.95	6.47	7.56

### HPLC method development and optimization

Extensive investigation into various stationary phases led to the selection of the C18 column. Columns of different particle sizes (5µ, 3µ) and lengths (250 mm, 100 mm) were used. Satisfactory results were obtained from using a C18, 4.5 × 150 mm, 3.6 μm column. Different water and acetonitrile ratios were used to obtain sharp, clear, and separate peaks for the analytes of interest. The optimal mobile phase mixture of water: acetonitrile (60:40, v/v) was determined, effectively avoiding the use of buffers and preventing salt formation and column clogging. Adjusting the pH using 0.1% Orthophosphoric acid (pH 2.5) improved the peak sharpness. The mobile phase was developed to decrease the organic phase ratio and increase the aqueous phase ratio to make the method simple, applicable, and reduce the cost. Using different flow rates, the flow rate of 1.5 mL/min enhanced the resolution while maintaining acceptable analysis time and achieving the best separation for the mixture. Wavelengths of 290 and 320 nm were the most convenient for qualitative and quantitative detection of the Remdesivir-Nitazoxanide and Daclatasvir-Piroxicam mixtures, respectively. For each mixture, there was no effect on purity or constant ratio in solvent or in plasma when equal ratios were mixed, as confirmed by HPLC analysis.

### HPLC assay validation

Method validation was performed at two levels: bioanalytical validation in plasma in accordance with ICH M10 (Guideline 2022b) using plasma calibration standards and quality control samples prepared by protein precipitation, and solution-based validation following ICH Q2(R2) (Guideline 2022a) using standard and pharmaceutical dosage form solutions. This dual approach confirms the suitability of the proposed method for both plasma quantification and pharmaceutical analysis.

### Selectivity and specificity

Specificity was evaluated by injecting the mobile phase (blank), individual working standard solutions, pharmaceutical dosage form samples, and a mixture of the working standards (Fig. . In addition, blank human plasma samples obtained from six independent sources were analyzed to assess potential matrix interference. As illustrated in Fig. 4, the developed method demonstrated high selectivity, producing distinct and well-resolved peaks for each analyte without interference from endogenous plasma components or co-eluting substances.

**Fig. 3:**
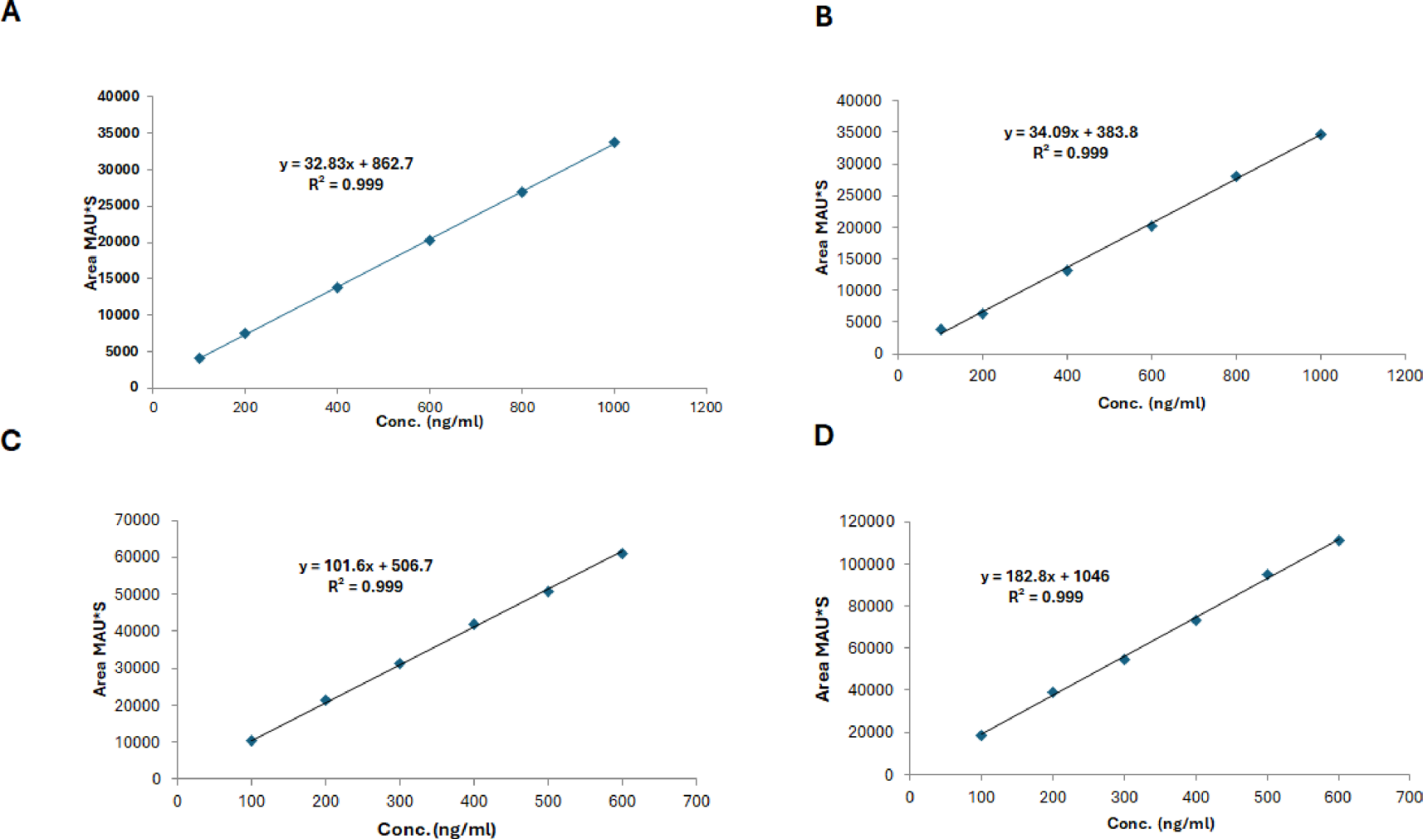
Linearity obtained from plasma samples of **A**, Remdesivir; **B**, Nitazoxanide; **C**, Daclatasvir; and **D**, Piroxicam with a 0.999 correlation coefficient

**Fig. 4:**
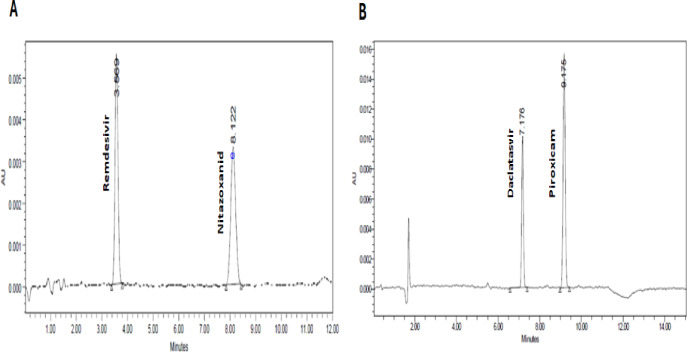
A chromatogram showing the retention time for **A**, Remdesivir-Nitazoxanide mixture, and **B**, Daclatasvir-Piroxicam mixture in plasma with concentration of 1000 ng/ml and 600 ng/ml, respectively

### Linearity and range

The linearity was assessed over defined concentration ranges for each analyte. For the Remdesivir-Nitazoxanide mixture, the linear range was 100 − 1000 ng/mL, while for the Daclatasvir-Piroxicam mixture, it was 100–600 ng/mL. The generated calibration curves exhibited excellent linearity, with correlation coefficients (r^2^) of 0.999 for all analytes. The respective calibration plots are shown in Fig. 3, and the linearity data are summarized in Table 4.

**Table 4:** Calibration parameters obtained from the HPLC method using serial dilution of Remdesivir, Nitazoxanide, Daclatasvir, and Piroxicam in plasma

Parameter	Remdesivir	Nitazoxanide	Daclatasvir	Piroxicam
Concentration Range (ng/mL)	100–1000	100–1000	100–600	100–600
Slope (b)	32.83	34.09	101.6	182.8
Intercept (a)	862.7	383.8	506.7	1046
Standard error of estimation	189.12	1044.44	856.924	1200.066
Linear regression equation	y = 32.83x + 862.7	y = 34.09x + 383.8	y = 101.6x + 506.7	y = 182.8x + 1046
Standard error of intercept	189.12	1044.44	856.924	1200.066
Confidence limit of intercept (t95%)	48.998–1676.488	1983.56–2751.26	3180.31–4193.77	4117.40–6209.53
Standard error of slope	0.3438	1.0004	2.2700	3.179
Confidence limit of slope (t95%)	31.356–34.315	29.792–38.401	91.865–111.399	169.146–196.502
Detection limit (LOD) (ng/mL)	16.34	53.252	27.824	21.665
Quantitation limit (LOQ) (ng/mL)	49.52	161.371	84.316	65.640
Correlation coefficient (r2)	0.999	0.999	0.999	0.999

### Limit of quantitation (LOQ) and limit of detection (LOD)

Limit of quantitation (LOQ) and limit of detection (LOD) were determined using the signal-to-noise (S/N) ratio method. Serial dilutions of Remdesivir, Nitazoxanide, Daclatasvir, and Piroxicam standards were prepared, and calibration curves were generated. The standard deviation of the y-intercept was used to calculate LOQ and LOD based on the following formulas:

LOQ = (σ / Slope) × 10.

LOD = (σ / Slope) × 3.3.

The calculated LOQ values were 49.52 ng/mL for Remdesivir, 161.371 ng/mL for Nitazoxanide, 84.316 ng/mL for Daclatasvir, and 65.640 ng/mL for Piroxicam. Corresponding LOD values were 16.34 ng/mL for Remdesivir, 53.25 ng/mL for Nitazoxanide, 27.82 ng/mL for Daclatasvir, and 21.66 ng/mL for Piroxicam. Detailed data is provided in Table 4.

### Accuracy

Method accuracy was evaluated through recovery studies at three concentration levels for each analyte. The accuracy for Remdesivir and Nitazoxanide was assessed at 200, 400, and 600 ng/mL, while the accuracy for Daclatasvir and Piroxicam was evaluated at 400, 500, and 600 ng/mL. Recovery percentages and relative standard deviation values (RSD%) were calculated for each concentration level. The RSD% values were consistently below 2%, indicating high method accuracy. Detailed recovery results are presented in Tables 5.

**Table 5:** Accuracy results of the proposed HPLC method

Parameter	Remdesivir	Nitazoxanide	Daclatasvir	Piroxicam
Concentration range (ng/mL)	100–1000	100–1000	300–600	300–600
Mean recovery	98.57	99.88	100.75	98.91
SD	1.49	1.58	1.45	1.33
RSD %	1.51	1.85	1.44	1.35
Accuracy*	98.57 ± 1.49	99.88 ± 1.58	100.75 ± 1.45	98.91 ± 1.33

SD, Standard Deviation; RSD%, Relative Standard Deviation Percentage

*Accuracy is presented as recovery mean ± standard deviation

**Table 6:** Assessment of the intra-day and inter-day precision of the proposed HPLC method

Drug	Concentration (ng/mL)	Intra-day		Inter-day	
		RSD%	Mean RSD	RSD%	Mean RSD
Remdesivir	600	0.835	1.90	0.80	1.86
	400	3.51	1.90	4.25	1.86
	200	1.38	1.90	0.52	1.86
Nitazoxanide	600	0.41	1.71	3.17	1.81
	400	2.89	1.71	0.36	1.81
	200	1.82	1.71	1.90	1.81
Daclatasvir	600	0.844	1.82	1.31	0.71
	500	3.318	1.82	0.74	0.71
	400	1.313	1.82	0.074	0.71
Piroxicam	600	0.84	1.77	1.16	0.48
	500	2.91	1.77	0.17	0.48
	400	1.48	1.77	0.11	0.48

*RSD%: Relative standard deviation percentage

#### Precision

Precision was assessed by evaluating intra-day and inter-day variability. Intra-day precision involved triplicate analyses of three concentration levels on the same day, whereas inter-day precision was determined by repeating the process over three consecutive days. Recovery percentages and RSD% values were calculated Table 6. The low RSD% values confirmed the precision and reproducibility of the method.

### Robustness

Robustness was tested by deliberately varying chromatographic conditions, including acetonitrile concentration (38–42%), mobile phase pH (± 0.2 units), and flow rate (1.3–1.7 mL/min). The resolution between Remdesivir, Nitazoxanide, Daclatasvir, and Piroxicam, as well as between these analytes and other matrix components, remained unaffected, demonstrating the robustness of the method.

## Discussion

Drug combinations are particularly effective in treating viral infections because they significantly reduce the likelihood of resistance against a single drug (Einav et al. 2010; Bobrowski et al. 2021). Moreover, the combined antiviral effect can be more potent than that of each drug individually, a phenomenon referred to as synergy. Some research indicates that antiviral drug combinations are more prone to exhibit synergistic effects if they belong to different classes, operate through distinct mechanisms, or target various stages of the viral life cycle (Bobrowski et al. 2021).

Our analysis focused on the FDA-approved antivirals Remdesivir and Daclatasvir, which were used in intensive care. The anti-SARS-CoV-2 activity of these drugs was tested alone and in binary combination (in equal concentrations) with FDA-approved antimicrobial (Nitazoxanide), and FDA-approved anti-inflammatory (Piroxicam), respectively. As revealed by HPLC, neither the purity nor the composition of any drug was affected in solvent or in plasma. We were able to confirm the previously reported synergistic anti-SARS-CoV-2 activity of the Remdesivir-Nitazoxanide mixture (Bobrowski et al. 2021; Stewart 2023). Remdesivir was initially developed to combat the Ebola virus during the 2014–2016 epidemic in West Africa (Siegel et al. 2017). It became the first FDA-approved antiviral treatment for COVID-19 (Shah et al. 2023). It is currently available under the name VEKLURY (Bakheit et al. 2023). Subsequently, other antiviral medications like Nirmatrelvir-Ritonavir (Paxlovid) and Molnupiravir (Lagevrio) also received FDA approval for use (Shah et al. 2023). Nitazoxanide has recently been explored for its potential use against SARS-CoV-2 due to its known anti-coronaviral effects. It was originally recognized as a promising candidate for antiviral drug repurposing against SARS-CoV-2. The drug is bioavailable, well-tolerated, with no significant adverse effects reported in healthy adults (Stockis et al. 2002; Rossignol 2016; Bobrowski et al. 2021). Enhanced anti-SARS-CoV-2 activity was also observed for the Daclatasvir-Piroxicam mixture. Daclatasvir is an anti-HCV NS5A inhibitor (Sacramento et al. 2021). Its potential role in treating COVID-19 has been previously reported (Sacramento et al. 2021; Shabani et al. 2021). Piroxicam is currently not recommended in the treatment of COVID-19 (Wojcieszyńska et al. 2022; Perico et al. 2023). However, some reports demonstrate potential benefits of Piroxicam against SARS-CoV-2 (Ricciotti et al. 2021; Varodi et al. 2022; Perico et al. 2023). Taken together, the results of the current study demonstrate the utility of drug repurposing and preclinical testing of drug combinations for the discovery of potential therapies for COVID-19.

Furthermore, we were able to develop a simple HPLC method for the concurrent detection of each analyte of the investigated binary mixtures, Remdesivir-Nitazoxanide mixture and Daclatasvir-Piroxicam mixture, in both pure form and in human plasma. The current method demonstrated high accuracy, precision, specificity, and robustness, fulfilling the stringent criteria required for pharmaceutical quality control applications.

## Conclusion

Enhanced anti-SARS-CoV-2 activity was observed for the investigated Remdesivir-Nitazoxanide and Daclatasvir-Piroxicam mixtures. As revealed by HPLC, neither the purity nor the composition of any drug was affected in solvent or in plasma. Further investigations are essential to demonstrate the anti-SARS-CoV-2 action of these mixtures in relevant mouse models and human clinical trials. In addition, a validated, robust, and straightforward HPLC method was successfully developed for the simultaneous determination of Remdesivir-Nitazoxanide and Daclatasvir-Piroxicam mixtures. The method demonstrated high accuracy, precision, specificity, and robustness, fulfilling the stringent criteria required for pharmaceutical quality control applications. The successful application of the current method for pharmaceutical dosage forms and detection of analyte residues in plasma underscores its potential for broader use in pharmaceutical and clinical settings. Furthermore, the method offers a sustainable and efficient alternative to more complex analytical procedures currently employed in the pharmaceutical industry.

## Electronic Supplementary Material

Below is the link to the electronic supplementary material.

Additional file 1.

Additional file 2.

## Data Availability

No datasets were generated or analysed during the current study.

## Abbreviations

CC_50_: Half maximum cytotoxic concentration _50_
COVID-19: Coronavirus disease
CPE: Cytopathic effect
DAA: Direct-acting antiviral
DMEM: Dulbecco’s modified eagle’s medium
FBS: Fetal bovine serum
FDA: Food and drug administration
HBV: Hepatitis B virus
HCV: Hepatitis C virus
HPLC: High performance liquid chromatography
HQC: High quality control
IC_50_: Half maximal inhibitory concentration _50_
LLOQ: Lower limit of quantification
LOD: Limit of detection
LOQ: Limit of quantitation
LQC: Low quality control
MERS-CoV: Middle East respiratory syndrome coronavirus
MOI: Multiplicity of infection
MQC: Mid quality control
NS5A: Nonstructural viral protein 5 A
NSAID: Nonsteroidal anti-inflammatory drug
OD: Optical density
P2: Passage 2
PDA: photodiode array
pen/strep: Penicillin/streptomycin
PFU: Plaque-forming unit
QC: Quality control
RSD%: Relative standard deviations
S/N: Signal-to-noise ratio
SARS-CoV-2: Severe acute respiratory syndrome coronavirus 2
SVR: Sustained virologic response
TCID50: Tissue culture infection dose-50
TPCK: DMEM containing 2% FBS, 2% pen/strep, and 1% L-1-tosylamido-2-phenylethyl chloromethyl ketone
