# Supplementary figures and images for: Anti-COVID-19 activity and simple HPLC method for concurrent detection of repurposed drugs in novel binary mixtures
Source: AMB Express. 2026 Apr 9;16:47. doi: 10.1186/s13568-026-02030-8 (PMC13087079; doi:10.1186/s13568-026-02030-8)

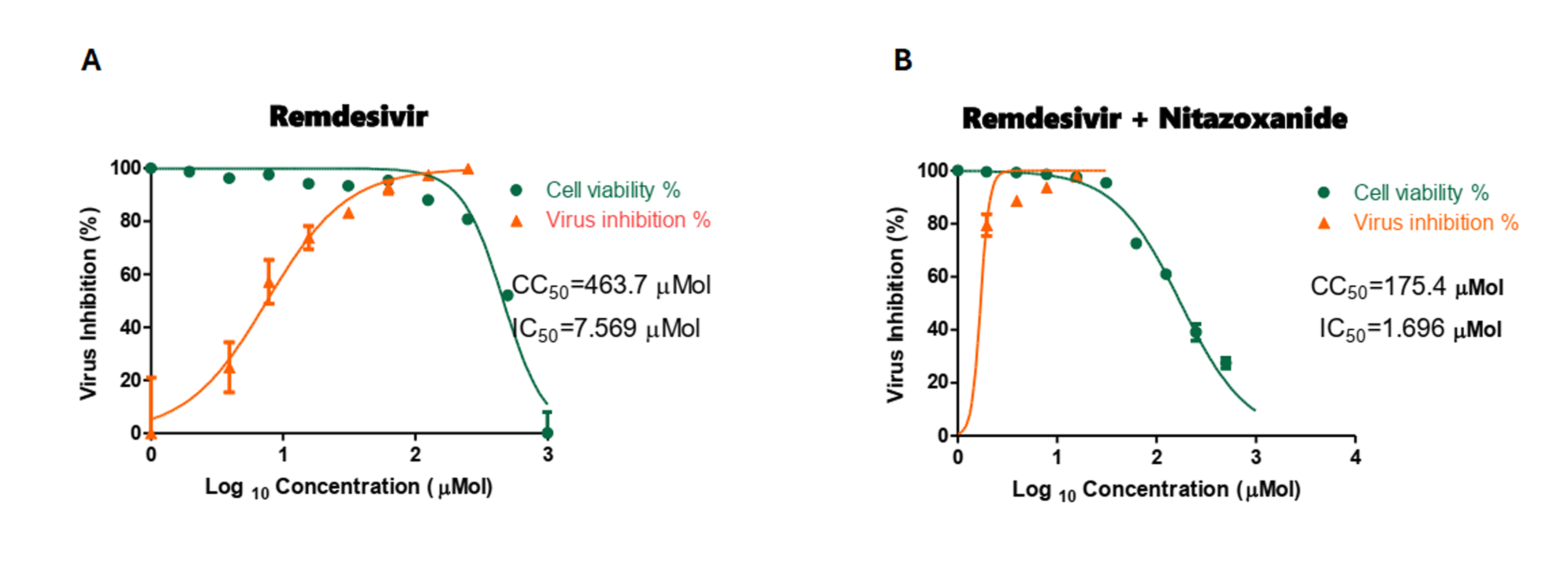

Supplement: Supplementary file 1 — Additional file 1. [file 13568_2026_2030_MOESM1_ESM.tif]

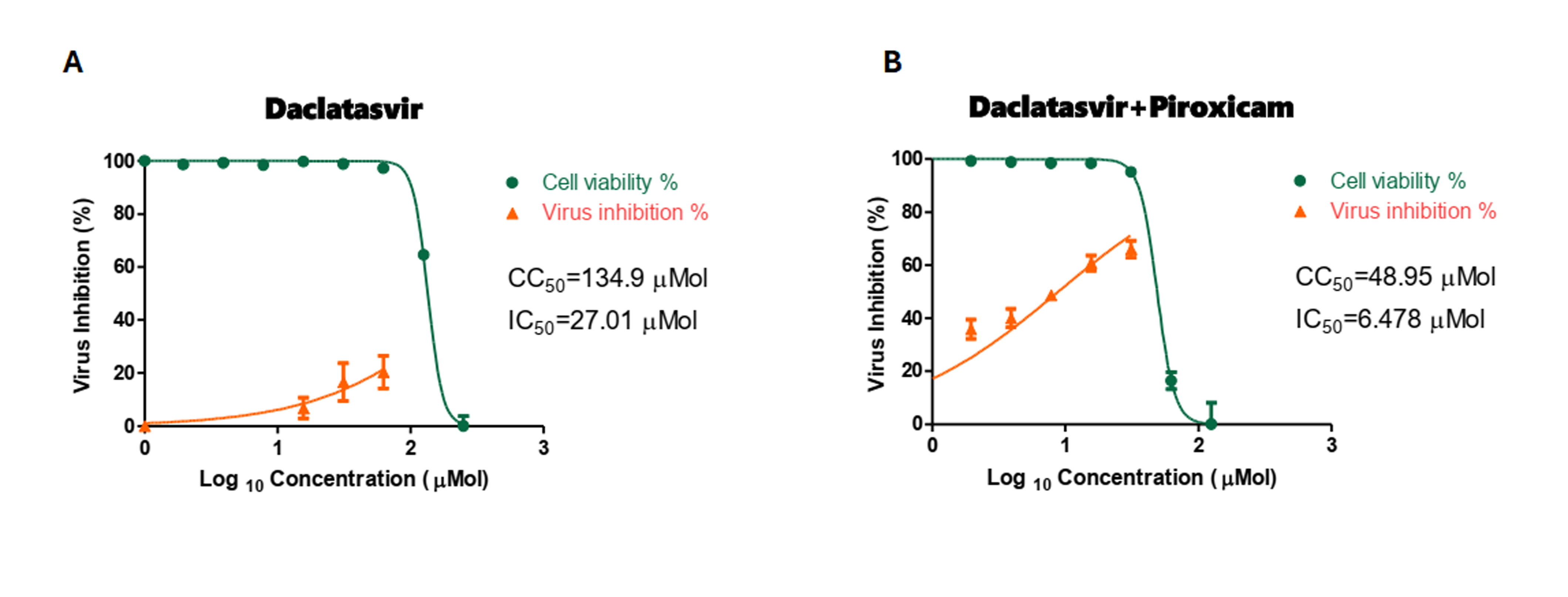

Supplement: Supplementary file 2 — Additional file 2. [file 13568_2026_2030_MOESM2_ESM.tif]
